# Trends of cancer incidence among Chinese older adults from 2005 to 2016: A log-linear regression and age-period-cohort analysis

**DOI:** 10.3389/fpubh.2022.1023276

**Published:** 2022-10-18

**Authors:** Hong Guo, Kangqian Lin, Kaiyue Yang, Zhenrong Ma, Miao Cao, Yunhua Hu, Yizhong Yan

**Affiliations:** Department of Public Health, Shihezi University School of Medicine, Xinjiang, China

**Keywords:** cancer, epidemiology, aged, age-period-cohort model, log-linear regression

## Abstract

**Background:**

To study the corresponding strategies for controlling cancer in older adults aged 60 and above in China, a comprehensive assessment of disease burden is required. Therefore, we will introduce the cancer epidemiological characteristics of older adults in China over a recent 12 year period.

**Methods:**

The age-period-cohort model was constructed using the cancer incidence data from the Chinese Cancer Registry Annual Report published in 2008–2019. The annual change percentage (APC) was estimated by log-linear regression to reflect the time trend. The data from the GLOBOCAN 2020 database was selected for worldwide comparative analysis.

**Results:**

The cancer incidence in older adults aged 60 and above in China showed a decreasing trend (APC = −0.73%, *P* = 0.009). The urban/rural ratio of cancer incidence increased from 0.94 to 1.07 (t = 3.52, *P* < 0.05), while the sex ratio (male/female) showed a significant decreasing trend only in rural areas (t = −6.77, *P* < 0.05), and the ratio decreased from 2.02 to 1.72. The results of the age-period-cohort model showed that the cancer incidence increased with age in both males and females, urban and rural areas. The RR of period effect increased from 2005 to 2010, then decreased from 2010 to 2015, and the downward trend was more obvious. The RR of the later-born cohort was lower than that of the earlier-born cohort in rural areas. Lung, gastric, colorectal, esophageal, liver, and breast cancers were common cancers in Chinese older adults. Lung cancer incidence ranked first in males, and it decreased with time in the 75–79 and 80–84 age groups (APC_75 − 79_ = −1.10%, APC_80 − 84_ = −0.88%, all *P* < 0.05). Breast cancer incidence ranked first among female in the 60–64 age group and showed an increasing trend (APC_60 − 64_ = 1.52%, *P* < 0.05).

**Conclusions:**

The cancer incidence in Chinese older adults aged 60 and above showed a decreasing trend, but it was still at a relatively high level. The key targets of prevention and treatment should be males, urban areas, younger people, older adults aged 60–69, lung, gastrointestinal, and breast cancers in the future.

## Introduction

Global aging is increasingly serious. Take France, which was the first to enter an aging society, as an example. In 2015, the number of people aged 60 and above in France exceeded that of 0–14 years. If fertility remains low, France's population will fall by 13.5% in 2050. With the reversal of the age structure of the French population, the median age will rise to an unprecedented age of 45–50, and France will enter the era of an inverted population pyramid (1). Japan, which is quite similar to China in social and cultural aspects, has an aging situation. It is estimated that in 2030, more than 40% of Japan's working population will be 60 or older. Long-term ultra-low fertility rate and rising life expectancy will lead to slow population decline and aging in these decades, and the growth of Gross Domestic Product (GDP) and per-capita GDP will slow down (2). Obviously, the impact of aging is not limited to changes in the proportion of older adults, but will reshape the age structure of various countries' populations and bring a series of social problems.

China has become an aging society, at an exponential rate. In the past decade, the aging rate of China has surpassed that of Japan (3). In 1999, older adults aged 60 above accounted for 10% of the total population, marking that China has officially entered an aging society (4). Since then, China has entered a period of rapid development of population aging. The number of people aged over 60 in China has reached 264 million in 2020, accounting for 19% of the total population (5). Meanwhile, it is estimated that by 2030, people aged 60 years or above will exceed 25% (6). As the population ages, society and families are facing significant issues. How to deal with the aging population and improve the quality of life of older adults has become a prominent topic in recent studies. According to the study, the cancer incidence in elders in China from 2006 to 2010 was 8.18 times that of the young and middle-aged groups, and the risk of cancer was also higher (7). Furthermore, in a review of the death causes of Chinese older adults between 1990 and 2019, cancer was the second leading cause (8). The prevention and control of cancers in older people have become one of the important public health problems in China.

Concurrently, China has issued policy documents such as the Healthy China Action (2019–2030) and Outline of the Healthy China 2030 Plan, which regard the prevention and control of cancers as one of the 15 key special actions, and older adults as one of the key groups. The purpose of this study was to construct an age-period-cohort model and estimate the APC by log-linear regression to conduct a comprehensive study on the epidemic characteristics of cancers among older adults aged 60 and above in China from 2005 to 2016, so as to provide a reference basis for the prevention and control of cancers in China.

## Methods

### Data sources

In this study, the cancer incidence data among older adults aged 60 and above in China from 2005 to 2016 were selected from the Chinese Cancer Registry Annual Report compiled by the National Cancer Center (NCC) published in 2008–2019 (9–20). During this period, the number of cancer registries in China increased from 45 in 2008 to 682 in 2019, with a coverage rate of 27.60%.

The regional distribution of this study was based on the classification criteria in the Chinese Cancer Registry Annual Report: cities above prefecture level were urban areas, and counties and county-level cities were rural areas.

The cancer incidence data in the world and countries with different Human Development Index (HDI) were selected from the GLOBOCAN 2020 database for comparative analysis (21).

### Analysis index

According to the international classification of diseases (ICD-10) (22), the incidence, age-specific incidence rate, constituent ratio (%), sex incidence ratio (male/female, SIR), regional incidence ratio (urban/rural, RIR), age-standardized rate (ASR), age-standardized incidence rate of the world (ASRW) and annual percent change (APC) of all cancers (C00–C96) was calculated.

APC was used to describe the trend of cancers in older adults for 12 years. The natural logarithm of the incidence rate (r) is represented by y, that is, y = ln(r). Then, with y as the dependent variable and x (year) as the independent variable, a regression linear model is fitted: *y* = α+β*x*+ε, where α is a constant term, β is a regression coefficient, and ε is a random error term.


$$\text{APC}=\left({e^{\beta }}^{\text{\_}}1\right)\times 100$$

If the trend of incidence does not change, that is, APC = 0 (invalid hypothesis), the statistical test of APC can be based on whether β is equal to 0 or not. The 95% confidence interval (CI) of APC is estimated to be:


$$\begin{array}{l} \text{Lower 95\%:}CI_{L}=\left\{e^{\left[\beta -\left(T\times SE_{\beta }\right)\right]}-1\right\}\times 100\\ \text{Upper 95\%:}CI_{U}=\left\{e^{\left[\beta +\left(T\times SE_{\beta }\right)\right]}-1\right\}\times 100 \end{array}$$

### Statistical methods

Stratification was carried out according to different regions (urban and rural), sex, age groups (60–64, 65–69, 70–74, 75–79, 80–84, and ≥85 years) and time (2005–2016). Assuming that the cancer cases in the population follow the Poisson distribution, the SAS statistical software Genmod module was used to fit the Poisson regression, with the log of the population as an offset variable, the dependent variable was the incidence, and the independent variables were sex, urban and rural. The annual RIR, SIR and its 95%CI (adjusted region and sex) were calculated, and the linear regression was used to test the ratio trend (23).

The time trend of cancer incidence was analyzed by APC and its 95%CI. With the log of cancer incidence as the dependent variable, the regression coefficient β of log-linear regression was used to estimate the APC (24, 25). “Increase” or “decrease” was used to describe a statistically significant trend.

The expression of the age-period-cohort model was as follows:


$$log\left(R\right)=log\left(P_{\text{ij}}\right)+u+\alpha Age_{\text{i}}+\beta Period_{\text{j}}+\gamma Cohort_{\text{k}}+\varepsilon$$

where *R* is the outcome variable of an event, such as incidence (/10^5^); *P* represents the population of age group i in the period group j; *u* stands for intercept term of incidence; α, β, γ represent age, period and cohort effect at all levels, respectively, and ε is residual. The relative risk (RR) is the exponential value of the coefficient (26, 27).

To avoid expanding the scope of the birth cohort and affecting the time accuracy of disease risk description, the age-specific data of 2005, 2010, and 2015 were selected to simulate the age-period-cohort model (28). For the collinearity problem existing in the classical age-period-cohort model, the intrinsic estimator approach was used to solve the collinearity problem in the age-period-queue model (29, 30), and the fitting superiority of the Comprehensive Evaluation Model based on the Akaike information criterion.

## Results

### Overall situation

In 2005–2016, there were 3,478,089 cases of cancers in Chinese people aged 60 and above, and the median incidence was 1,057.56/10^5^ (range, 997.89/10^5^ ~ 1,123.32/10^5^). The cancer incidence showed a decreasing trend during this period (APC = −0.73%, *P* = 0.009) (Table 1).

**Table 1:** Cancer incidence in Chinese older adults aged 60 and above from 2005 to 2016.

**Year**	**Cases (*n*)**	**Incidence (/10^5^)**	**ASR(/10^5^)**
2005	84,147	1,039.43	1,004.46
2006	96,575	1,087.75	1052.70
2007	97,769	1,078.10	1,042.60
2008	119,136	1,123.32	1,078.89
2009	144,946	1,092.14	1,061.15
2010	207,618	1,085.56	1,060.95
2011	244,493	1,069.09	1,050.52
2012	331,244	1,049.22	1,035.94
2013	390,588	1,044.14	1,035.12
2014	501,211	1,021.30	1,014.71
2015	567,881	997.89	992.34
2016	692,481	998.73	995.53
APC (%)	-	−0.73	−0.39
95%CI (%)	-	(−1.23, −0.23)	(−0.84, 0.07)
*t*	-	−3.23	−1.90
*P*	-	0.009	0.087

APC, annual change percentage; CI, confidence interval.

In 2016, a total of 692,480 cases occurred. Among them, the proportion of each age group was: 23.6% for 60–64 years (163,727 cases), 21.7% for 65–69 years (149,994 cases), 18.5% for 70–75 years (128,171 cases), 16.6% for 75–79 years (115,125 cases), 12.3% for 80–84 years (84,953 cases), and 7.3% for ≥ 85 years (50,510 cases). The cancer cases of males accounted for 61.0% (422,399 cases), that of females accounted for 39.0% (270,081 cases). The proportion for urban and rural areas were 53.9% (373,176 cases) and 46.1% (319,304 cases), respectively.

### Sex-specific

The cancer incidence showed a decreasing trend in males and females, and the trend was more obvious in males (APC_males_ = −1.00%, APC_females_ = −0.63%, all *P* < 0.05). For different regions, the cancer incidences of males and females in urban were stable (all *P* > 0.05), however, that of rural males showed a decreasing trend (APC = −2.10%, *P* < 0.05) (Table 2). The SIR of older adults in China and urban areas was stable (all *P* > 0.05). However, the SIR in rural areas decreased from 2.02 in 2005 to 1.72 in 2016 (*P* < 0.05) (Table 3).

**Table 2:** The APC of cancer incidence in Chinese older adults aged 60 and above with different characteristics from 2005 to 2016 (%).

**Age**	**Urban areas**			**Rural areas**			**All areas**		
	**M**	**F**	**B**	**M**	**F**	**B**	**M**	**F**	**B**
60–64	1.45^a^	1.75^a^	1.61^a^	−2.45^a^	−0.59	−1.81^a^	0.46	0.98^a^	0.70^a^
65–69	1.08^a^	0.07	0.73^a^	−1.05^a^	−0.01	−0.65^a^	0.68^a^	−0.10	0.45^a^
70–74	−0.61	−1.14^a^	−0.82^a^	−2.96^a^	−1.67^a^	−2.26^a^	−1.06^a^	−1.49^a^	−1.16^a^
75–79	−1.33^a^	−0.64	−1.08^a^	−2.30^a^	−0.62^a^	−1.30^a^	−1.60^a^	−0.96^a^	−1.29^a^
80–84	−1.21^a^	0.25	−0.48	−1.64^a^	−0.10	−0.55	−1.57^a^	−0.23	−0.85^a^
≥85	−1.61	0.28	−0.45	−0.53	0.68	0.55	−1.93^a^	−0.21	−0.84^a^
Total	−0.48	−0.35	−0.32	−2.10^a^	−0.79	−1.48^a^	−1.00^a^	−0.63^a^	−0.73^a^

APC, annual change percentage; M, males; F, females; B, both of the males and females.

^a^*P* < 0.05.

**Table 3:** The sex (male/female) incidence ratio in Chinese older adults aged 60 and above from 2005 to 2016.

**Year**	**Urban areas**		**Rural areas**		**All areas**	
	**SIR**	**95%CI**	**SIR**	**95%CI**	**SIR**	**95%CI**
2005	1.63	(1.60, 1.65)	2.02	(1.96, 2.08)	1.71	(1.69, 1.74)
2006	1.67	(1.64, 1.69)	1.94	(1.88, 2.00)	1.72	(1.70, 1.74)
2007	1.62	(1.59, 1.64)	1.92	(1.87, 1.97)	1.68	(1.66, 1.70)
2008	1.63	(1.60, 1.65)	1.84	(1.79, 1.89)	1.66	(1.64, 1.68)
2009	1.62	(1.60, 1.64)	1.76	(1.73, 1.80)	1.66	(1.64, 1.67)
2010	1.68	(1.66, 1.70)	1.82	(1.79, 1.84)	1.72	(1.70, 1.73)
2011	1.67	(1.65, 1.68)	1.83	(1.80, 1.85)	1.72	(1.71, 1.74)
2012	1.61	(1.60, 1.63)	1.81	(1.79, 1.83)	1.70	(1.68, 1.71)
2013	1.61	(1.59, 1.62)	1.76	(1.75, 1.78)	1.68	(1.67, 1.69)
2014	1.60	(1.58, 1.61)	1.72	(1.71, 1.74)	1.65	(1.64, 1.66)
2015	1.57	(1.56, 1.59)	1.71	(1.70, 1.72)	1.64	(1.63, 1.65)
2016	1.61	(1.60, 1.62)	1.72	(1.70, 1.73)	1.66	(1.65, 1.66)
*t*	−2.00		−6.89		−2.15	
*p*	0.074		< 0.001		0.057	

SIR, sex incidence ratio; CI, confidence interval.

### Region-specific

**Figure 1:**
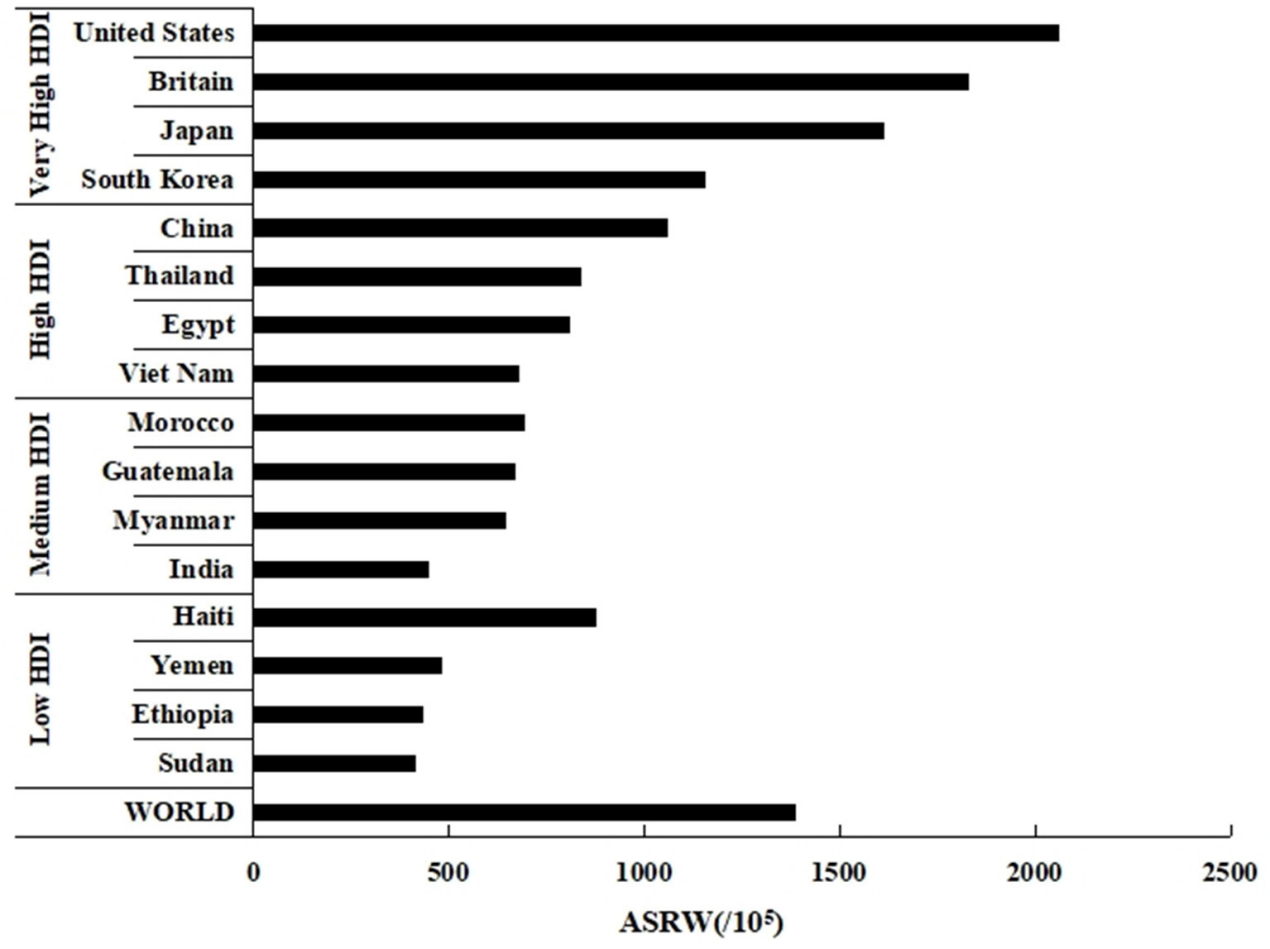
Estimated age-standardized incidence rates of cancers in older adults aged 60 and above in countries with different HDI in 2020 (Date source: GLOBOCAN 2020, https://gco.iarc.fr/today/).

According to the data of the GLOBOCAN2020 database, the cancer incidences among older adults in United States, Britain, Japan, South Korea and China were 1,386.4/10^5^, 2,061.0/10^5^, 1,829.7/10^5^, 1,615.0/10^5^, 1,156.8/10^5^, 1,060.2/10^5^, respectively. The cancer incidence in Chinese older adults was lower than that in countries with very high HDI and the world average level (Figure 1).

The cancer incidence among older adults in urban areas was stable (*P* > 0.05), while in rural areas it showed a decreasing trend (APC = −1.48%, *P* < 0.05) (Table 2). In 2005, the RIR was 0.94 among Chinese older adults. By 2016, the RIR increased to 1.07 (*P* < 0.05). The RIR among females was stable (*P* > 0.05). However, the RIR among males increased from 0.86 in 2005 to 1.04 in 2016 (*P* < 0.05) (Table 4).

**Table 4:** The regional (urban/rural) incidence ratio in Chinese older adults aged 60 and above from 2005 to 2016.

**Year**	**M**		**F**		**B**	
	**RIR**	**95%CI**	**RIR**	**95%CI**	**RIR**	**95%CI**
2005	0.86	(0.85, 0.88)	1.07	(1.04, 1.10)	0.94	(0.93, 0.96)
2006	0.88	(0.86, 0.90)	1.02	(0.99, 1.05)	0.93	(0.92, 0.95)
2007	0.87	(0.86, 0.89)	1.04	(1.01, 1.06)	0.93	(0.92, 0.95)
2008	0.95	(0.93, 0.97)	1.07	(1.05, 1.10)	0.99	(0.98, 1.01)
2009	1.06	(1.05, 1.08)	1.16	(1.13, 1.18)	1.09	(1.08, 1.10)
2010	1.05	(1.03, 1.06)	1.13	(1.12, 1.15)	1.07	(1.06, 1.08)
2011	0.99	(0.98, 1.00)	1.08	(1.06, 1.09)	1.02	(1.01, 1.02)
2012	1.00	(0.99, 1.01)	1.12	(1.11, 1.13)	1.04	(1.04, 1.05)
2013	1.00	(0.99, 1.01)	1.10	(1.09, 1.11)	1.04	(1, 03, 1.04)
2014	1.03	(1.03, 1.04)	1.12	(1.11, 1.13)	1.06	(1.05, 1.07)
2015	1.02	(1.02, 1.03)	1.11	(1.10, 1.12)	1.05	(1.05, 1.06)
2016	1.04	(1.04, 1.05)	1.11	(1.10, 1.12)	1.07	(1.06, 1.07)
*t*	3.73		2.19		3.49	
*p*	0.004		0.053		0.006	

RIR, regional incidence ratio; CI, confidence interval; M, males; F, females; B, both of the males and females.

### Age-period-cohort analysis

Age effect: among the Chinese older adults aged 60 and above, whether males or females, urban or rural areas, the cancer incidence increased first and then decreased with age, reaching a peak in the 80–84 age group.

Period effect: for males and females, the RR increased from 2005 to 2010, then decreased from 2010 to 2015, and the downward trend was more obvious. The RR in rural areas decreased from 2005 to 2015.

**Figure 2:**
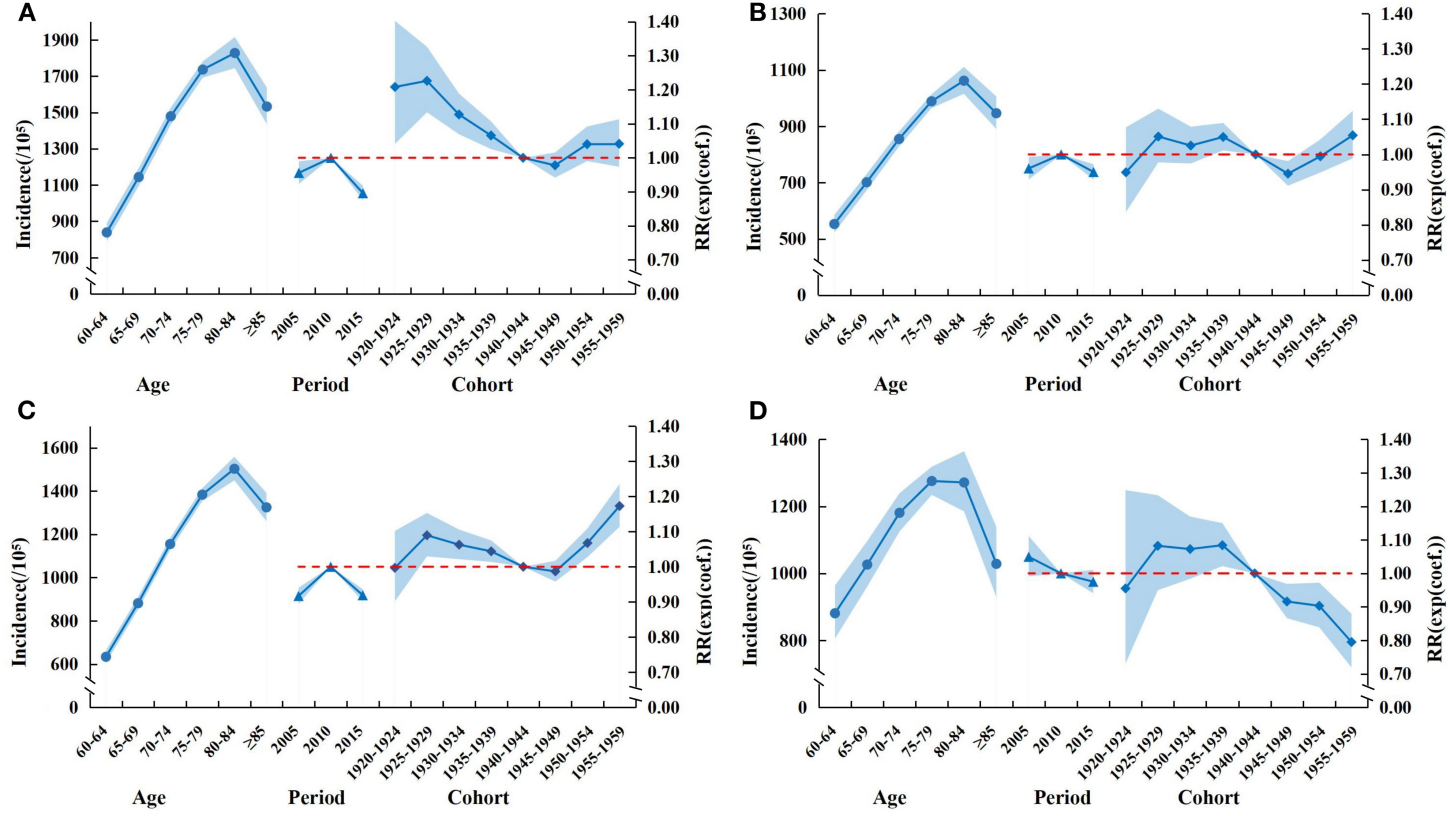
Age-period-cohort model of cancer incidence in Chinese older adults aged 60 and above from 2005 to 2016. **(A)** Male. **(B)** Female. **(C)** Urban. **(D)** Rural.

Cohort effect: for males, females, and urban areas, the RR decreased in birth-cohorts born from 1935 to 1949 and increased in birth-cohorts born from 1950 to 1959. In rural areas, the RR deceased in birth-cohorts born from 1935 to 1959 (Figures 2A–D).

### Cancer composition

**Figure 3:**
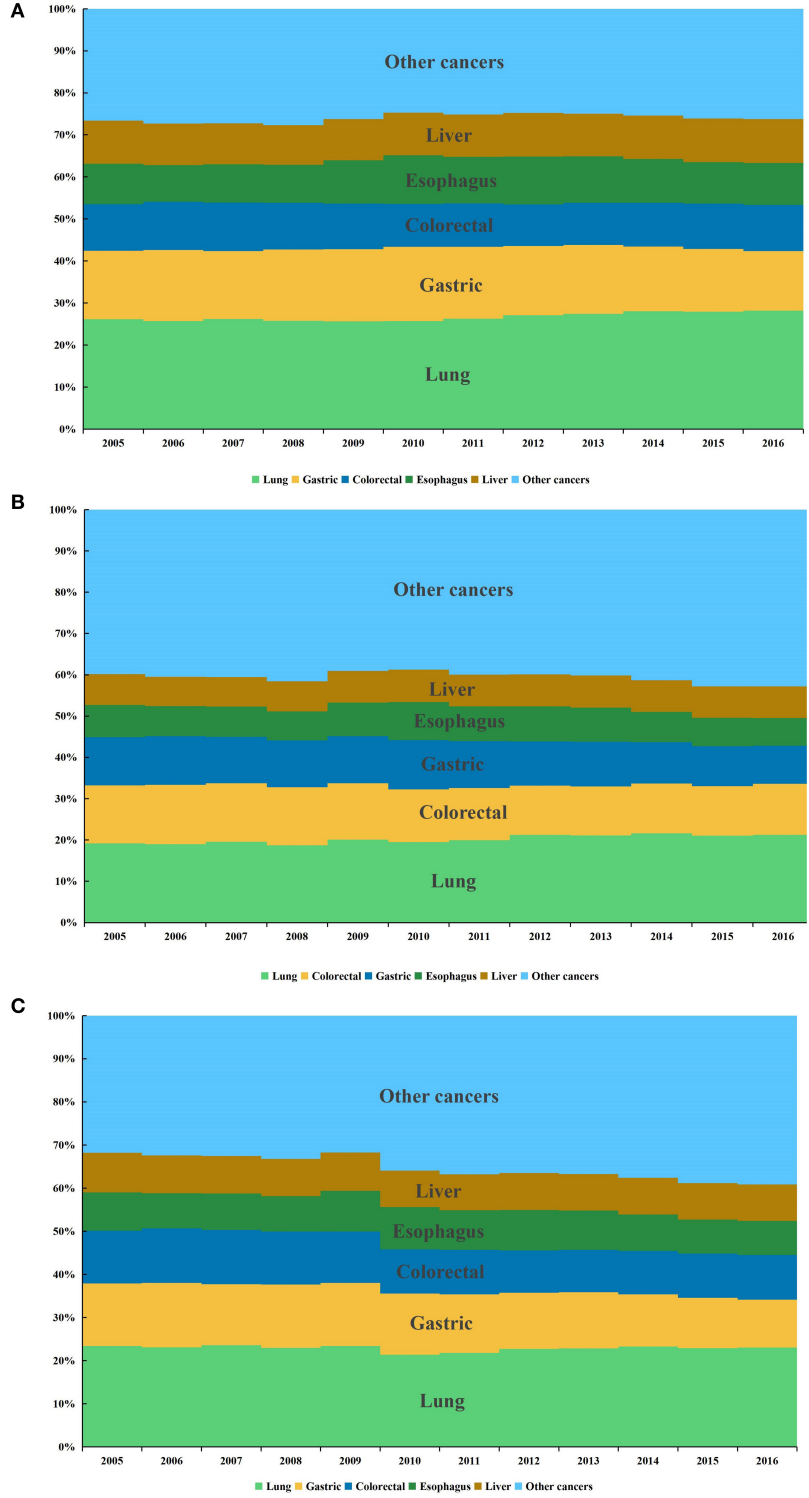
Composition of cancers in Chinese older adults aged 60 and above from 2005 to 2016. **(A)** Male. **(B)** Female. **(C)** Both.

Lung cancer ranked first among Chinese older adults from 2005 to 2016, with a total of 844,286 cases, and followed by gastric, colorectal, esophageal, liver, breast, prostate, pancreatic, bladder cancers and lymphoma, the top 10 cancer cases accounted for about 81.6% of all cancers. Similarly, for males and females, the lung cancer ranked first, with 567,608 cases and 276,678 cases, respectively. It was followed by gastric, colorectal, esophageal, liver, prostate, bladder, pancreatic, lymphoma and oral cavity and pharynx cancer in males. The top 10 cancer cases accounted for 89.1% of all male cancers. In females, the incidence of lung cancer was followed by those of colorectal, gastric, breast, esophageal, liver, pancreatic, gallbladder, brain and nervous system, corpus uteri and uterus unspecified cancer, the top 10 cancers accounted for about 80.1% of all cancers (Figures 3A–C).

### Time trend of the top five cancers in different sexes and ages

**Figure 4:**
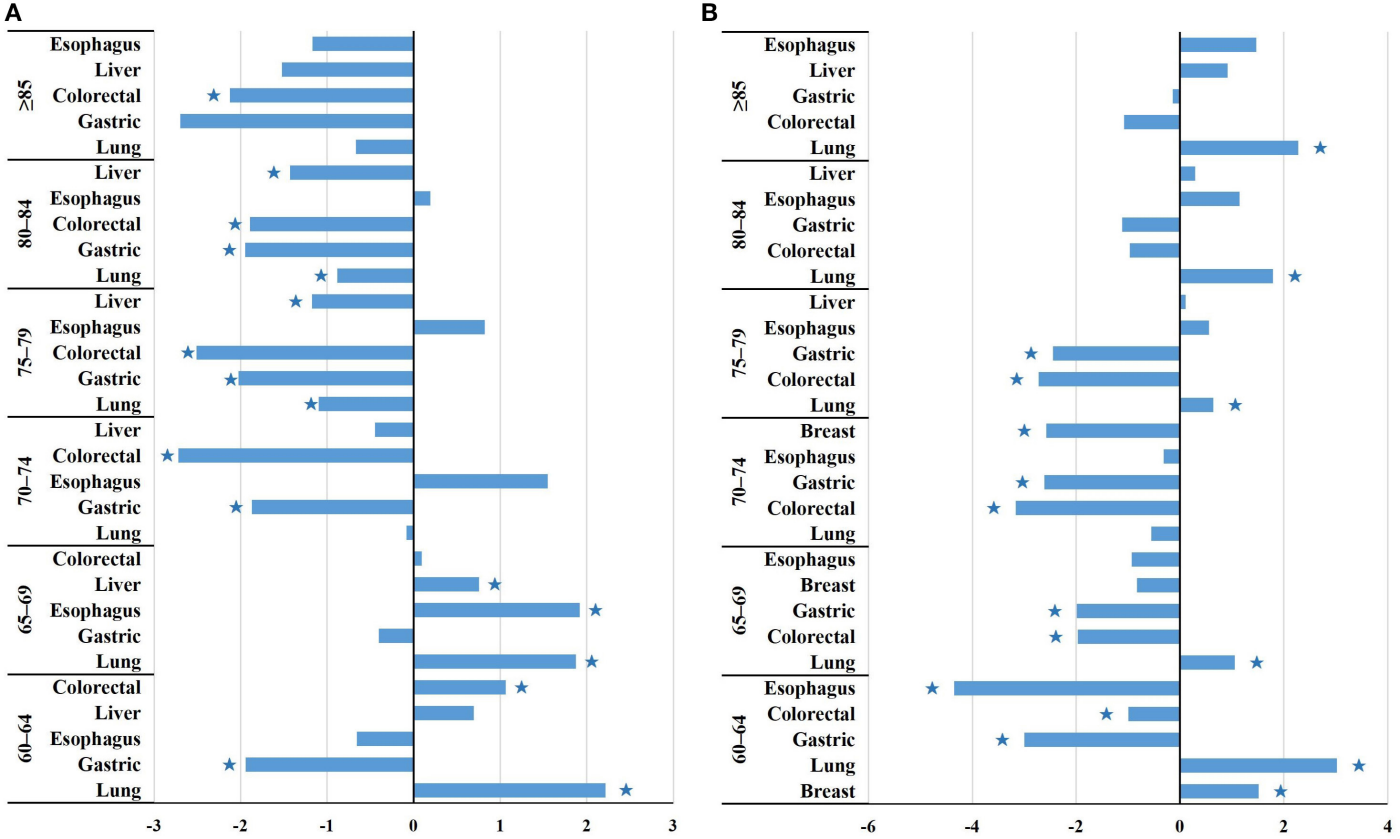
The changing trend of the incidence of the top five cancers among Chinese older adults aged 60 and above of different genders and different age groups from 2005 to 2016. ^*^P < 0.05. **(A)** Male. **(B)** Female.

For males: lung cancer incidence ranked first in all age groups and it increased with time in 60–64 and 65–69 age groups (APC_60 − 64_ = 2.22%, APC_65 − 69_ = 1.87%, all *P* < 0.05), while it decreased in 75–79 and 80–84 age groups (APC_75 − 79_ = −1.10%, APC_80 − 84_ = −0.88%, all *P* < 0.05). Gastric cancer incidence showed a decreasing trend in 60–64, 70–74, 75–79, and 80–84 age groups (APC_60 − 64_ = −1.94%, APC_70 − 74_ = −1.87%, APC_75 − 79_ = −2.02%, APC_80 − 84_ = −1.95%, all *P* < 0.05). Colorectal cancer incidence increased in 60–64 age group (APC_60 − 64_ = 1.07%, *P* < 0.05), while it decreased in 70–74, 75–79, 80–84 and ≥85 age groups (APC_70 − 74_ = −2.72%, APC_75 − 79_ = −2.51%, APC_80 − 84_ = −1.89%, APC_≥85_ = −2.12%, all *P* < 0.05). Liver cancer incidence increased in 65–69 age group (APC_65 − 69_ = 0.76%, *P* < 0.05), while it decreased in 75–79 and 80–84 age groups (APC_75 − 79_ = −1.18%, APC_80 − 84_ = −1.43%, all *P* < 0.05) (Figure 4A).

For females: breast cancer ranked first in 60–64 age group and increased with time (APC_60 − 64_ = 1.52%, *P* < 0.05), while it decreased in 70–74 age group (APC_70 − 74_ = −2.58%, *P* < 0.05). Lung cancer incidence ranked first among older females except for the 60–64 age group and showed an increasing trend in 60–64, 65–69, 80–84 and ≥85 age groups (APC_60 − 64_ = 3.02%, APC_65 − 69_ = 1.06%, APC_80 − 84_ = 1.79%, APC_≥85_ = 2.28%, all *P* < 0.05). Gastric cancer incidence decreased in 60–64, 65–69, 70–74 and 75–79 age groups (APC_60 − 64_ = −3.00%, APC_65 − 69_ = −1.99%, APC_70 − 74_ = −2.61%, APC_75 − 79_ = −2.45%, all *P* < 0.05). Similar to gastric cancer, colorectal cancer decreased in 60–64, 65–69, 70–74 and 75–79 age groups (APC_60 − 64_ = −0.99%, APC_65 − 69_ = −1.97%, APC_70 − 74_ = −3.16%, APC_75 − 79_ = −2.73%, all *P* < 0.05). Esophagus cancer incidence showed a decreasing trend in 60–64 age group (APC_60 − 64_ = −4.35%, *P* < 0.05) (Figure 4B).

## Discussion

Based on the cancer incidence data collected by the NCC from 2005 to 2016, the present study analyzed the cancer epidemic characteristics in Chinese older adults aged 60 years and above over a recent 12 year period. The results showed a decreasing trend in the cancer incidence of older adults from 2005 to 2016, but the incidence was still at a high level. The most economical strategy to reduce the cancer incidence should be primary prevention. The Chinese government has implemented a number of health promotion initiatives, including the national cancer prevention and control plan (1986–2000 years) and the programme of cancer prevention and control in China (2004–2010 years) (31, 32). The reduction in cancer incidence among Chinese older adults may be consistent with the implementation of these initiatives.

Numerous studies have shown that smoking may increase the risk of more than 15 cancers, including lung, esophageal, gastric and liver cancer (33, 34). China has been making efforts to reduce tobacco use, including increasing fees and outlawing smoking in enclosed public spaces (35). Although the benefits of these initiatives have started to be felt, they are insufficient. Studies have shown that in 2019, the smoking prevalence in China was 24.3%, of which 49.7% for male and 3.54% for female. Compared with 32.7% of the global male smoking prevalence, the prevalence of Chinese male smoking was at a higher level, which was also significantly higher than that in very high HDI countries, such as the United States (19.9%), Britain (21.7%) and Japan (33.4%) (36).

However, this study found that the cancer incidence in Chinese older adults was lower than that in countries with very high HDI, such as the United States, Britain, South Korea, Japan. Some studies found that the smoking-caused respiratory and circulatory diseases [Respiratory and circulatory diseases accounted for 85.1% of Chinese older adults suffering from chronic diseases in 2016 (37), and the prevalence was higher than that in countries with very high HDI (8, 38, 39)] may reduce the proportion of Chinese older adults to accept cancer screening and their willingness to seek medical advice (40, 41), this phenomenon could mask the true cancer incidence of Chinese older adults. For example, we known that a national cancer screening program was launched by the Chinese government in 2005, covering eight common cancers, including lung, breast and colorectal cancer (42). But studies have shown that people with comorbidities were 0.79, 0.62, and 0.93 times likely to be screened for lung, breast and colorectal cancer than those without chronic conditions, respectively (43, 44). And a study of lung cancer patients showed that patients with chronic obstructive pulmonary disease (COPD) took twice as long to consult with lung cancer symptoms (45). Therefore, China should continue to take strong measures to control the smoking prevalence in order to reduce the occurrence of respiratory and other diseases, increase the likelihood of participating in cancer screening, and reduce the disease burden.

In addition, the cancer incidence among older people in a country is also related to factors such as the number and life expectancy of older adults (46, 47). The relatively lower life expectancy in China compared to countries with very high HDI may also contribute to the low cancer incidence in Chinese older adults (48). In 2019, the coverage rate of China's cancer registry was 27.60%, while as of July 2019, the coverage rate of the US cancer registration system was 96%, and that of the United Kingdom and South Korea was close to 100% (49). Incomplete coverage of cancer registries may lead to the low incidence among Chinese older adults. Therefore, improving the construction of China's cancer registration system, making full use of big data and new information technology and achieving accurate monitoring of cancers are prerequisites for understanding the epidemic trend of cancers among Chinese older adults (50).

From the perspective of the differences between urban and rural areas in China, the cancer incidence among older adults in rural areas was decreased, while the incidence in urban areas was stable. There has been a reversal of the incidence ratio between urban and rural areas in China. In 2005, the incidence in rural areas was 6% higher than that in urban areas; by 2016, the incidence in urban areas was 7% higher than that in rural areas. This may be due to differences in living habits, eating habits, and environmental pollution between urban and rural areas (51). The large number of rural migrant elderly people choosing to seek medical treatment in cities and the growing awareness of cancer prevention among rural residents may also contribute to this phenomenon (52–54). For example, China launched a major national health care reform project “early diagnosis and early treatment of cancer in rural areas” in 2009 and launched screening work for high-risk groups such as lung cancer in rural areas (55). Moreover, the decreasing trend in cancer incidence among rural older adults was mainly reflected in male with more serious morbidity, and the sex ratio dropped from 2.02 in 2005 to 1.72 in 2016. This may be related to the cancer control strategy taken for male high-risk groups in China.

Aging is a risk factor for cancer. Theoretically, the cancer incidence increases with age. Countries with very high HDI levels, such as the United States and Japan, have shown this trend (56, 57). However, the results of this study showed that the cancer incidence in older people aged 85 years and above was lower than that in the 75–79 and 80–84 groups, which may be due to the competitive death of diseases such as the circulatory and respiratory systems after 80 years of age (8). This may also be due to the decrease in the possibility of seeing a doctor in the group aged ≥ 85 years, which could lead to missed diagnosis (58).

The cohort effect is regarded as the interaction between age growth and period change, which shows that after suffering specific historical events, the change of exposure risk factors affects all age groups at the same time, and different birth cohorts often have different risks (59). In this study, whether male or female, urban or rural areas, the RR decreased in birth-cohorts born from 1935 to 1949. It indicated that people born during this period experienced a lower risk of cancer. Environmental improvements may reduce the risk of cancer (60). With China's industrial reforms in 1920 (61), air pollution began to improve, and environmental change could have a lasting impact on people born between 1935 and 1949, which may promote cancer prevention. Therefore, environment management should be continued to prevent cancer.

In terms of the cancer composition, lung, gastric, and colorectal cancers were the main cancers that threaten the health of Chinese older adults, among which lung cancer ranked first in both male and female. The top 10 cancers in Chinese older adults were the same as those in countries with very high HDI, but the order was different. China had a high incidence of lung cancer and digestive system cancers, such as liver, gastric, and esophageal cancers, whereas countries with very high HDI in Europe and the United States had a high incidence of thyroid, breast, and prostate cancers (62–64). The difference between the cancer spectrum in China and that in countries with very high HDI has caused a serious disease burden in China. China should expand the screening of cancers and vigorously perform health education to reduce the incidence of related cancers among older adults. Lung cancer showed an increasing trend among Chinese older females except the 70–74 and 75–79 age groups, however, it decreased among males aged 75–79 and 80–84 age groups. The different trends may be related to the change in the concept of smoking, the decrease in smoking prevalence in males with age, and the increase in females (65, 66). Females' exposure to second-hand smoke, outdoor air pollution and the use of indoor solid fuels for heating and cooking may also contribute to an increased incidence of lung cancer (67, 68). Breast cancer ranked first in older females aged 60–64 years, and showed an increasing trend, suggesting that older females of 60–64 years should be considered as the key object of prevention and treatment of breast cancer. Although the incidence of gastric cancer and colorectal cancer in Chinese older adults showed an overall decreasing trend, gastrointestinal cancers still had a high incidence. Screening and treatment of gastrointestinal cancers should be strengthened to reduce the disease burden.

In summary, the cancer incidence in Chinese older adults showed a decreasing trend, but it was still high. The incidence of Chinese older males was higher than that of females, and the incidence of urban elderly was higher than that in rural areas. The cancer incidence increased with age in both males and females, urban and rural areas. The RR of the later-born cohort was lower than that of the earlier-born cohort in rural areas. In the future, China should regard males, urban, and low-age elderly as the key population for the prevention and treatment of cancer, and focus on the control of lung, gastrointestinal, breast, and other major cancers that threaten the health of Chinese older adults. In addition, more attention should be paid to prevention and treatment of cancer in the younger population, which may reduce the cancer risk in the old age and may even reduce the cancer incidence in the population as a whole.

## Data availability statement

The original contributions presented in the study are included in the article/supplementary files, further inquiries can be directed to the corresponding authors.

## Author contributions

YY, HG, and KL initiated the study. HG, KL, KY, ZM, and MC collected and processed the data. HG and KL performed the statistical analysis, visualization, and drafted the manuscript. YH and YY revised the manuscript. All authors read, contributed to the framework construction, result interpretation, manuscript revision, and approved the final version of the manuscript.

## Funding

This study was funded by the Shihezi University High-Level Talent Project (No. RCZK2021B28) and the Shihezi Science and self-Funded project of ShiHezi University (No. ZZZC202125).

## Conflict of interest

The authors declare that the research was conducted in the absence of any commercial or financial relationships that could be construed as a potential conflict of interest.

## Publisher's note

All claims expressed in this article are solely those of the authors and do not necessarily represent those of their affiliated organizations, or those of the publisher, the editors and the reviewers. Any product that may be evaluated in this article, or claim that may be made by its manufacturer, is not guaranteed or endorsed by the publisher.

## Abbreviations

GDP: Gross Domestic Product
NCC: National Cancer Center
HDI: Human Development Index
APC: annual change percentage
SIR: sex incidence ratio
RIR: regional incidence ratio
ASR: age-standardized rate
ASRW: age-standardized incidence rate of the world
CI: confidence interval
RR: relative risk
M: males
F: females
B: both of the males and females.
